# Innovative multimodal DOTA/NODA nanoparticles for MRI and PET imaging for tumor detection

**DOI:** 10.1186/2197-7364-1-S1-A80

**Published:** 2014-07-29

**Authors:** Charles Truillet, Penelope Bouziotis, Charalambos Tsoukalas, Lucie Sancey, Franck Denat, Frédéric Boschetti, Dimitris Stellas, Constantinos D Anagnostopoulos, Vassilis Koutoulidis, Lia A Moulopoulos, François Lux, P Perriat, Olivier Tillement

**Affiliations:** ILM, UMR 5306, University of Claude Bernard Lyon 1, 69622 Villeurbanne Cedex, France; Matériaux Ingénierie et Science, INSA Lyon, CNRS, University of Lyon, 69622 Villeurbanne, France; Radiochemistry Studies Laboratory, Institute of Nuclear and Radiological Sciences and Technology, Energy and Safety, National Center for Scientific Research “Demokritos”, Athens, Greece; Institut de Chimie Moléculaire de l’Université de Bourgogne, UMR CNRS 6302, University of Bourgogne, 21078 Dijon Cedex, France; CheMatech, 21000 Dijon, France; Department of Cancer Biology, Biomedical Research Foundation, Academy of Athens, Athens, Greece; Center for Experimental Surgery, Clinical and Translational Research, Biomedical Research Foundation, Academy of Athens, Athens, Greece; Department of Radiology, University of Athens Medical School, Areteion Hospital, Athens, Greece

The knowledge of the exact tumor stage is essential to adapt therapeutic strategies or to follow the evolution of the tumor after therapy in order to increase the survival chance. The multi-tasking diagnostics that combine techniques such as PET and MRI could really improve imaging tumor stage. PET mainly offers functional information about the disease with high sensitivity. MRI offers predominantly morphological information, able to provide an excellent soft tissue contrasts due to its high resolution.

In this setting, we propose a polysiloxane nanoplatform gadolinium-based (AGuIX^®^) inferior to 5 nm able to combine PET/MRI imaging for tumor detection [1]. The size of this multimodal platform allows a good renal clearance [2].

The nanoparticles are composed of silica matrix functionalized by gadolinium chelate (DOTAGA-Gd), MRI positive contrast agent and free chelate (DOTAGA and NODAGA) for radiolabelling with isotopic radioactive such as ^64^Cu and ^68^Ga [3]. ^68^Ga isotope is an excellent candidate for nuclear imaging because of its availability. ^64^Cu has a long lifetime and can be used for Brachytherapy. Physical and chemical properties of the AGuIX^®^ coupled with 2,2',2''-(10-(2,6-dioxotetra hydro-2H-pyran-3-yl)-1,4,7,10-tetraazacyclododecane-1,4,7-triyl)triacetic acid (DOTAGA) or 2,2'-(7-(1-carboxy-4-((2,5-dioxopyrrolidin-1-yl)oxy)-4-oxobutyl)-1,4,7-triazonane-1,4-diyl) diacetic acid (NODAGA) were finely characterized.

Due to the high specificity of the NODAGA chelator with those radioisotopes, the purity of radiochemical assessment of the radiolabeled AGuIX^®^ was found superior to 98% in agreement with required purity regulations (>95%). First dual images on healthy animal in vivo data in a mouse model show both MR and PET localization of probe within the kidneys since the elimination was renal. MR and PET images on animal with tumor showed that the nanoparticles were able to achieve passive targeting in mice bearing tumors.

The nanoparticles AGuIX^®^ exhibit a real potential for **multimodal imaging** that should permit the PET/MRI clinical investigations with a unique nanoparticle.
